# The mediating role of psychological resilience and coping styles among Chinese medical students in the relationship between perceived social support and learning engagement among medical students

**DOI:** 10.3389/frcha.2026.1835372

**Published:** 2026-08-21

**Authors:** Yue Li, Fan Zhang, Mengran Zhang, Hua Zhu, Zhe Jin

**Affiliations:** 1 Department of Social Medicine and Health Management, Shenyang Medical College, Shenyang, China; 2 Department of Clinical Medicine, Xingtai Medical College, Xingtai, China; 3 School of Medical and Health, Weifang Business Vocational College, Weifang, China; 4 Infection Control Department, Baotou Central Hospital, Baotou, China

**Keywords:** chain mediating effect, learning engagement, medical student, perceived social support, positive coping style, psychological resilience

## Abstract

**Objective:**

To explore the relationship between college medical students' Perceived Social Support and learning engagement and the mediating role of psychological resilience and coping styles.

**Methods:**

The Perceived Social Support Scale (PSSS), the Simple Medical Coping Style Questionnaire (SCSQ), Ego-resiliency Scale (ER89), and the Undergraduate Work Engagement Scale (UWES-S) were selected to conduct a questionnaire survey of 2,375 medical students.

**Results:**

The analysis showed that there was a significant positive relationship between perceived social support, psychological resilience, positive coping styles and learning engagement(*r* = 0.274∼0.973,*P* < 0.01);The results of the mediated effects test indicated that the mediated paths of perceived social support affecting learning engagement through psychological resilience and positive coping styles and their chain mediated effects all showed statistical significance respectively (*β* = 0.083,0.033,0.071).

**Conclusions:**

Medical students' perceived social support not only affects learning engagement directly, but also indirectly through the mediating role of psychological resilience and positive coping style and the chain mediating role of psychological resilience-positive coping style.

## 1 Introduction

In today's world, medical education requires medical students not only to master excellent clinical skills but also to cultivate high professional ethics and a passion for lifelong learning (1). Because medical education is closely linked to future careers, many universities have strengthened and implemented measures to foster an academic culture that encourages students to study diligently, increase the time they devote to their studies, and shift the focus of higher education quality assessment from outcomes to the learning process (2). Learning engagement is an extension of the concept of work engagement into the realm of learning; it refers to a state of behavior characterized by positive emotions and high energy levels demonstrated during the learning process (3), encompassing three dimensions: vitality, dedication, and focus (4). Researchers have found (5, 6) that increasing learning engagement can effectively improve the learning experience and enhance learning outcomes. Learning engagement positively predicts academic performance and achievement; it serves as a core indicator for assessing the quality of higher education and the development of medical students, and is closely related to the quality of healthcare services these students will provide in the future. With the development of the healthcare sector and the growing health needs of individuals, the importance placed on the professional competence of healthcare personnel is also increasing (7). As most medical students are future healthcare professionals and a strong reserve force for future healthcare services, there is an urgent need to study medical students' learning engagement and its influencing factors to provide theoretical and practical foundations for improving the quality of medical education.

Adolescence is a unique stage of life, beginning with sexual maturation and characterised by cognitive, emotional and social maturation, ultimately leading to a transition towards independence and adulthood (8). Due to the indefinable nature of adolescence, scholars currently find it difficult to conceptualize, study, and reasonably interpret it in a unified manner. This study posits that adolescence is characterized by a series of physical developmental features and long-term neural changes, and defines the age range as 14–24 years (9). The group of medical students examined in this study—aged 18–24—fully meets the current academic definition of “extended adolescence,” and medical students in this age group are particularly vulnerable to difficulties when facing academic pressures and challenges (10). therefore, it is necessary to support and promote the healthy development of adolescents, particularly in the areas of education and career.

Perceived social support refers to an individual' s expectations and subjective evaluation of social support (10); adequate external support can help students adapt to and adjust to their academic environment, allowing them to devote more time and energy to their studies (11). According to social exchange theory (12), the attention, recognition, and sense of trust that individuals gain from social support can foster close emotional bonds, which in turn translate into motivation for learning. This, in turn, increases their commitment to learning, leading to better performance in their academic lives (13).

With the development of positive psychology (14), the role of positive psychological resources in the process of individual growth and development has received widespread attention (15). High levels of stress in medical education lead to negative effects on medical students, such as decreased attention, anxiety and depression, deteriorating interpersonal relationships, and aversion to studying. These are often regarded as risk factors for psychological distress among medical students (16, 17); however, many students experiencing the same level of stress do not develop related psychological problems, and these students are considered to possess psychological resilience (18). Psychological resilience refers to an individual's “capacity for endurance” to actively cope with adverse events, recover quickly from adversity, and achieve positive development (19). Research indicates that perceived social support positively predicts psychological resilience (11), and strong psychological resilience enables effective coping with complex learning environments and positively influences academic engagement (20); Positive coping styles, as a form of self-stabilizing factor, refer to a state of maintaining optimism, seeking change, and seeking support when facing stress (21). Research indicates that an individual's perceived social support influences the choice of coping strategies (22). When people receive sufficient social support, they face problems with a positive attitude, enjoy better health, maintain positive emotions under stress, adopt appropriate coping strategies, and enhance their academic engagement (23). Previous research indicates that psychological resilience influences positive coping strategies (24). Students with psychological resilience often possess positive psychological traits such as optimism, emotional stability, and perseverance; they tend to adopt positive approaches to find positive meaning in the problems they face, demonstrate unique differences in physiological adaptive capacity when coping with stress, and achieve good adaptation (25). On the other hand, individuals who adopt positive coping strategies successfully resolve difficulties in their academic and personal lives by tapping into their inner potential and mobilizing internal and external protective factors, thereby indirectly influencing academic engagement (26).

Although previous studies have focused on medical students' academic engagement (13, 23, 26), existing studies on mediating effects are largely limited to a single mediating variable. Research on the sequential mediating role of psychological resilience and positive coping strategies remains insufficient; this may reveal a gradual psychological development pathway (27) that warrants further in-depth investigation and explanation. This study aims to analyze the underlying psychological mechanisms through which perceived social support influences medical students' academic engagement, thereby enriching the empirical research in this field.

Based on this, this study proposes three hypotheses: Perceived social support influences medical students' academic engagement; psychological resilience and positive coping strategies mediate the relationship between perceived social support and academic engagement, respectively; and psychological resilience and positive coping strategies exert a chained mediation effect on the relationship between perceived social support and academic engagement.

## 2 Materials and methods

### 2.1 Participants and procedure

From May 8th to 15th, 2024, an anonymous questionnaire survey was conducted among freshmen to seniors at a medical college in Shenyang. The survey employed stratified cluster sampling, stratified by four grades, with classes as the smallest sampling unit. At least 5–6 classes were selected from each grade to ensure that the number of sampled respondents was no less than 200 in each grade. After undergoing unified training, counselors clearly explained the research purpose, content, and precautions to the students and guided them in completing the questionnaire. A total of 2,400 questionnaires were distributed, and 2,375 valid questionnaires were collected, with a valid response rate of 98.96%. This study was approved by the Review Committee of Shenyang Medical College, and all survey participants provided informed consent.

### 2.2 Measures

#### 2.2.1 Perceived social support scale (PSSS)

The “Perceived Social Support Scale” revised by Jiang Qianjin (28, 29) was adopted. The scale consists of 12 items, covering family support, friend support, and other support. It adopts a Likert 7-point scoring system, ranging from “completely disagree” to “completely agree”, with scores ranging from 1 to 7. The total score ranges from 12 to 84, with higher scores indicating that the individual receives more social support. In this study, the Cronbach's *α* coefficient of this scale was 0.929.

#### 2.2.2 Simplified coping style questionnaire (SCSQ)

This scale was developed by Xie Yaning in 1998 based on the characteristics of the Chinese population (30, 31). In this study, we selected the positive coping subscale (12 items) and used a Likert 4-point scoring system, ranging from “not adopting” to “often adopting” with scores of 0–3, for a total score of 0–36. The higher the score, the stronger the positive coping ability. The Cronbach's *α* coefficient for this subscale in this study was 0.929.

#### 2.2.3 Ego-resiliency Scale (ER89)

ER89 was developed by BLOCK (32, 33) and other researchers (34, 35) based on the theory of psychological resilience. This scale employs a Likert 4-point scoring system, with “1” indicating “strongly disagree” and “4” indicating “strongly agree”. It consists of 14 items, with a total score ranging from 14 to 56. A higher score indicates a higher level of psychological resilience. This scale has a solid theoretical foundation, with simple and understandable items and a small number of items. It is often used in combination with other scales. In this study, the Cronbach's *α* coefficient for this scale was 0.874.

#### 2.2.4 Utrecht work engagement scale-student (UWES-S)

This scale was carefully developed by Schaufeli (36, 37) and later revised by Fang Laitan, Shi Kan, and others (38, 39). It consists of 17 items, covering three dimensions: vigor, dedication, and focus. Using the Likert 5-point scoring system, “completely disagree” is scored as 1 point, “completely agree” is scored as 5 points, and the total score ranges from 17 to 85. A higher score indicates greater engagement in learning. The Cronbach's *α* coefficient of the scale is 0.825, indicating good reliability.

### 2.3 Statistical analysis

Data were analyzed using SPSS 26.0 and AMOS 24.0 (40). For quantitative data, normality and homogeneity of variance tests were conducted first. If the data met the normality and homogeneity of variance tests, they were described using mean ± standard deviation ($\bar{x}\pm s$). Count data were described using proportion (*%*) composition. Difference analysis was conducted using *t*/*F* tests, and Pearson correlation analysis was used to explore the correlation between variables (41). The Harman's one-way *ANOVA* method was employed for the common method bias test. The non-parametric percentile Bootstrap method was employed for 5,000 repeated samplings to test whether the mediating effect was significant. The estimated effect size and 95% confidence interval were considered statistically significant with *P* < 0.05. A structural equation model was constructed using AMOS 24.0 software for verification.

## 3 Results

### 3.1 Common method bias test

The results of the Harman one-factor test (42) indicate that the unrotated principal component analysis identified seven factors with eigenvalues greater than 1. The first factor explained 34.59% of the variance, which is below the 40% threshold, suggesting that there is no significant common method bias in this study.

### 3.2 Participant characteristics

Among the respondents, there were 656 males (27.62%) and 1,719 females (72.38%); their average age was 18–24 (20.71 ± 1.88) years; 977 were freshmen (41.14%), 702 were sophomores (29.56%), 367 were juniors (15.45%), and 329 were seniors (13.85%); 978 were urban students (41.18%), and 1,397 were rural students (58.82%). See Table 1 for details.

**Table 1:** A comparison of scores on social support, psychological resilience, positive coping strategies, and learning engagement among medical students with different characteristics ($\bar{x}\pm s$).

Variable	*n*	PSSS		Psychological Resilience		Positive Coping Strategies		Learning Engagement	
		Scores	*t/F*	Scores	*t/F*	Scores	*t/F*	Scores	*t/F*
Sex			−7.242***		−2.030*		−4.268***		−2.368*
Male	656	55.35 ± 17.58		39.79 ± 9.84		34.16 ± 8.92		64.83 ± 15.78	
Female	1,719	60.56 ± 14.89		40.58 ± 8.03		35.66 ± 7.12		66.31 ± 12.65	
Grade			5.951***		0.586		0.501		0.401
Freshman	977	60.63 ± 5.18^b^^,^^c^^,d^		40.34 ± 8.07		35.37 ± 7.23		65.54 ± 13.03	
Sophomore	702	58.34 ± 14.96^a^		40.61 ± 8.24		35.05 ± 7.46		66.30 ± 12.03	
Junior year	367	57.43 ± 16.56^a^		40.08 ± 9.32		35.02 ± 8.24		66.11 ± 14.49	
Senior year	329	56.86 ± 17.49^a^		40.75 ± 8.57		35.52 ± 8.01		66.04 ± 14.91	
Place of origin			1.777		2.654**		1.487		0.500
Town	978	59.81 ± 16.64		40.92 ± 8.89		35.53 ± 8.01		65.78 ± 13.21	
Rural	1,397	58.63 ± 15.25		39.97 ± 8.33		35.05 ± 7.45		66.06 ± 14.14	

* *P* < 0.05.

** *P* < 0.05.

*** *P* < 0.001.

a indicates *P*<0.05：Compared to Freshman.

b indicates *P*<0.05：Compared to Sophomore.

c indicates *P*<0.05：Compared to Junior year.

d indicates *P*<0.05：Compared to Senior year.

### 3.3 Comparison of scores on social support, psychological resilience, positive coping strategies, and learning engagement among medical students with different characteristics

The study found that female students scored significantly higher than male students on the PSSS, ER89, positive coping strategies, and UWES-S scales. Urban students scored higher on the ER89 scale than rural students, and first-year students scored significantly higher on the PSSS scale than students in other years. All differences were statistically significant (*P* < 0.05), as shown in Table 1.

### 3.4 Descriptive statistics and correlation analysis

The results revealed significant positive correlations between pairs of the four variables—social support, psychological resilience, positive coping strategies, and academic engagement(*r* = 0.27–0.97, *P* < 0.01), as shown in Table 2.

**Table 2:** Descriptive statistics and correlation analysis.

Variable	1	2	3	4	5	6	7	8	9	10
1. PSSS	1									
2. Family Support	0.956**	1								
3. Friends' Support	0.970**	0.884**	1							
4. Other Support	0.969**	0.881**	0.925**	1						
5. Psychological Resilience	0.529**	0.499**	0.520**	0.513**	1					
6. Positive Coping Strategies	0.556**	0.523**	0.546**	0.540**	0.802**	1				
7. Learning Engagement	0.336**	0.318**	0.324**	0.330**	0.387**	0.400**	1			
8. Vitality	0.365**	0.348**	0.355**	0.355**	0.380**	0.402**	0.959**	1		
9. Dedication	0.319**	0.304**	0.307**	0.313**	0.376**	0.390**	0.973**	0.916**	1	
10. Focus	0.284**	0.266**	0.274**	0.283**	0.361**	0.363**	0.955**	0.851**	0.901**	1
$\bar{x}$	59.12	19.85	19.87	19.40	40.36	35.24	65.89	23.80	19.36	22.73
*s*	15.84	5.56	5.42	5.43	8.57	7.68	13.60	4.90	4.28	4.95

** *P* < 0.01.

### 3.5 Mediation effect analysis

After considering the influence of demographic factors such as gender, age, and grade, the chain mediation effect of psychological resilience and positive coping strategies between perceived social support and learning engagement among medical students was tested using model 6 of the SPSS macro program Process developed by Hayes (43). The research indicates that perceived social support significantly affects learning engagement (*β* = 0.126, *t* = 6.495***), and positively influences psychological resilience and positive coping strategies (*β* = 0.533, *t* = 30.319***; *β* = 0.178, *t* = 12.602***). Psychological resilience can effectively influence positive coping strategies (*β* = 0.706, *t* = 50.503***), and both psychological resilience and positive coping strategies positively predict learning engagement (*β* = 0.156, *t* = 4.944***; *β* = 0.189, *t* = 5.872***). The results are presented in Table 3.

**Table 3:** Hierarchical regression analysis of Variable relationships in mediational models.

Recurrence relation		Overall fit index		*F*	Significance of the regression coefficient	
Outcome variable	Predictor variables	*R*	*R* ^2^		*β*	*t*
Psychological Resilience	PSSS	0.535	0.284	158.071***	0.533	30.319***
Positive Coping Strategies	PSSS	0.818	0.670	685.464***	0.178	12.602***
	Psychological Resilience				0.706	50.503***
Learning Engagement	PSSS	0.440	0.194	71.121***	0.126	6.495***
	Psychological Resilience				0.156	4.944***
	Positive Coping Strategies				0.189	5.872***

*** *P* < 0.001.

### 3.6 Chain mediation effect analysis

**Figure 1:**
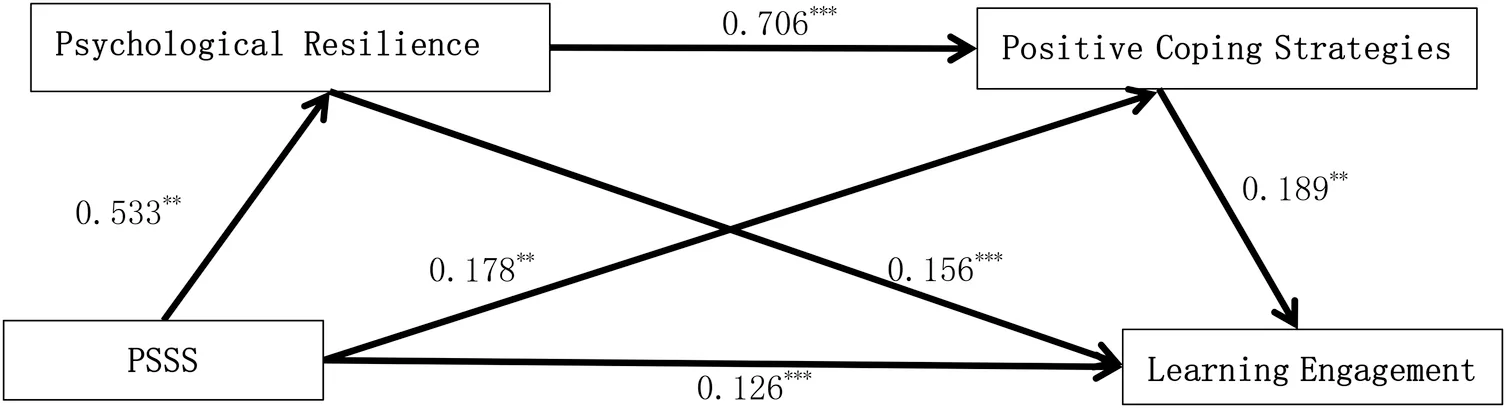
Diagram of the chain mediation effect model. This figure 1 was created by the first author.

The results showed that the direct effect of perceived social support on academic engagement, the mediating effects of psychological resilience and positive coping strategies on the relationship between perceived social support and academic engagement, and the chained mediating effect of psychological resilience and positive coping strategies all reached statistical significance, as shown in Table 4. Psychological resilience accounted for 28.92% of the mediating effect, positive coping strategies accounted for 11.50%, and the chained mediating effect accounted for 24.74%. The indirect effect accounted for 56.10%, while the direct effect accounted for 43.90%. Based on these data, the authors developed the mediating effect model presented in this section (Figure 1).

**Table 4:** An analysis of the chain mediation effects of psychological resilience and positive coping strategies.

Path	effect size	standard error	95% CI	Proportion of effect size (*%*)
Psychological Resilience (a1b1)	0.083	0.019	(0.044,0.123)	28.92%
Positive Coping Strategies (a2b2)	0.033	0.006	(0.020,0.048)	11.50%
Psychological Resilience and Positive Coping Strategies (a1a3b2)	0.071	0.016	(0.038,0.105)	24.74%
Direct effect (c')	0.126	0.019	(0.088,0.165)	43.90%
Indirect effects	0.161	0.016	(0.128,0.194)	56.10%
Overall effect	0.287	0.016	(0.255,0.320)	100.00%

Finally, validation using AMOS 24.0 confirmed the validity of this model; the fit statistics are presented in Table 5.

**Table 5:** Fitting statistics for the chained mediation model.

Indicator	*χ*^2^/*df*	RMSEA	GFI	AGFI	NFI	IFI
Criteria	<5	<0.05	>0.9	>0.9	>0.9	>0.9
Mediator model	3.756	0.034	0.994	0.986	0.997	0.998

## 4 Discussion

Medical curricula are highly challenging and involve relatively heavy academic workloads, requiring medical students to devote more time and effort (44). The results of the mediation analysis confirm that social support received during the learning process serves as an important protective factor for medical students' academic engagement. In other words, the more social support medical students perceive, the higher their level of academic engagement; establishing a multidimensional social support system can significantly improve academic engagement levels. According to the Resource Conservation Theory (45), changes in external and internal resources determine mental health status. Social support can compensate for the depletion of psychological resources caused by long-term academic stress, providing a theoretical explanation for how social support is transformed into internal psychological resources. Numerous studies have pointed out (11, 13) that support from friends and parents provides students with sufficient resources to cope with academic difficulties (46), thereby enhancing their academic engagement and enabling them to master solid medical professional skills. Therefore, providing medical students with adequate social support can enhance their academic engagement and help them acquire solid medical professional skills.

Previous studies (22, 24) have confirmed the association between psychological resilience and positive coping. Psychological resilience and positive coping strategies, acting as independent mediating variables, are influenced by perceived social support and, in turn, have a positive impact on academic engagement. According to the dynamic model of psychological resilience (47), adolescents have needs such as self-esteem, challenges, potential, and value during their development (19). If they can obtain sufficient support, understanding, and care from family, society, and school, this will help them develop a strong will and resilient traits. Research indicates that the more social support an individual perceives, the more conducive it is to developing a strong inner drive, enhancing psychological resilience, and promoting greater focus on studies (11). This is explained by the fact that both objectively available social support resources and subjectively experienced social support collectively constitute the internal and external protective factors of psychological resilience, thereby fostering psychological stability (48). At the same time, external support and psychological resilience can, to a certain extent, mitigate the impact of adverse situations, maintain consistency between inner thoughts and actions, and promote the development of strong adaptive abilities among medical students in both their academic and daily lives (49). This has a positive impact on their academic engagement, which in turn effectively reflects an individual's level of psychological resilience. Therefore, enhancing psychological resilience and fostering strong adaptive abilities can improve medical students' levels of academic engagement.

This study found that a positive coping style plays a partial mediating role in the relationship between perceived social support and academic engagement, acting as a “bridge.” When medical students with low levels of perceived social support face academic challenges, they often believe they are unable to overcome these difficulties (50). Conversely, if they adopt positive coping strategies, they can courageously accept challenges, continuously tap into their own potential, draw on favorable resources from all sources, mobilize protective factors, comprehensively explore diverse solutions to efficiently complete academic tasks, maintain proper learning motivation, rekindle their inner passion for learning, and enhance their academic engagement (45–47)—all of which are key to medical students achieving outstanding academic performance.

Positive coping strategies are crucial for enhancing academic engagement (26). In particular, medical students who lack sufficient social support need to focus on developing the ability to adopt positive and optimistic approaches to problem-solving, thereby fostering motivation and improving academic performance. Results indicate that psychological resilience plays a significant role in the selection of coping strategies (24, 51), and there is a significant positive correlation between psychological resilience and positive coping strategies. Medical students with low perceived social support can still engage effectively in their studies if they possess strong psychological resilience and adopt positive coping strategies. Having adequate social support resources is a key prerequisite for medical students to develop a high level of psychological resilience. A high level of psychological resilience not only fosters positive emotional states, enhances a sense of control over academic tasks, and significantly boosts enthusiasm for learning, but also helps medical students establish a healthy self-concept and develop excellent character traits (52, 53). Consequently, when facing challenges, they can fully utilize internal and external resources, adopt positive and reasonable measures to address problems, and exhibit sound psychological and behavioral patterns. Research indicates that individuals with high levels of psychological resilience are better able to perceive external support, mitigate maladaptive coping strategies and stress responses, and tend to adopt more positive coping strategies and behaviors when facing setbacks (24, 51, 52, 54), thereby promoting effective coping mechanisms. This is particularly true when rapidly adapting to a completely new learning environment during online learning. Therefore, with adequate social support, a high level of psychological resilience, and positive coping strategies, it is recommended that medical students increase their commitment to their studies to improve efficiency.

This study found that the overall learning engagement of medical students is at a relatively high level, which is consistent with the findings of Shida Q's survey (55). There is also a significant gender difference, with female students having an advantage, which is consistent with previous research findings (56), indicating that most medical students are relatively strict with themselves. Perceived social support has a significant positive impact on learning engagement, which is consistent with Xu Z's research findings on vocational students in China (57). Therefore, it is necessary for schools to cultivate classroom enthusiasm and enhance learning interest, as well as strengthen guidance and support in learning, so that medical students can more firmly commit to the medical profession.

This study has several limitations. First, there are limitations regarding the sample: the study surveyed only students at a single medical college in Shenyang, resulting in sampling bias (58). The grade composition within the sample obtained through stratified random sampling differs from that of the population (59), and there is room for improvement in terms of sample diversity and sample size; Second, there are limitations in variable selection (60). The study only examined the mediating roles of psychological resilience and positive coping strategies, failing to fully account for other important variables that may influence academic engagement (61); finally, academic engagement is a dynamic and evolving process, and the use of a cross-sectional design (62) limits the ability to accurately assess its developmental trajectory (56).

In future research, the sample size could be expanded and combined with qualitative interviews; subsequent studies should continue to examine the influence of other variables on the relationship between social support and medical students' academic engagement; longitudinal tracking, experimental methods, or case studies could be conducted to further refine the research; and more advanced and scientific statistical methods and theoretical models should be incorporated—from research design to data analysis—to explore other factors influencing academic engagement (63), while also considering the bidirectional interactions among variables.

## 5 Conclusion

Based on a theoretical hypothesis model, this study empirically examined the relationship between perceived social support and medical students' learning engagement, as well as its underlying mechanisms. All proposed hypotheses were validated (with each path coefficient *P* < 0.05). Specifically, the direct effect of perceived social support on learning engagement was significant. Both the independent mediating effect of psychological resilience and positive coping styles, as well as their chained mediating effect, reached a statistically significant level. The chained mediating effect accounted for 24.74% of the total effect, the indirect effect accounted for 56.10%, and the direct effect accounted for 43.90%. It is recommended that universities collaborate with families to improve the social support and security system for medical students, carry out targeted mental health interventions, enhance psychological resilience levels, and guide them to develop positive stress coping patterns, thereby effectively improving the overall quality and level of medical students' learning engagement.

## Data Availability

The raw data supporting the conclusions of this article will be made available by the authors, without undue reservation.
